# Genome characterization and mutation analysis of human influenza A virus in Thailand

**DOI:** 10.5808/gi.21077

**Published:** 2022-06-30

**Authors:** Somruthai Rattanaburi, Vorthon Sawaswong, Pattaraporn Nimsamer, Oraphan Mayuramart, Pavaret Sivapornnukul, Ariya Khamwut, Prangwalai Chanchaem, Kritsada Kongnomnan, Nungruthai Suntronwong, Yong Poovorawan, Sunchai Payungporn

**Affiliations:** 1 Interdisciplinary Program of Biomedical Sciences, Graduate School, Chulalongkorn University, Bangkok 10330, Thailand; 2 Research Unit of Systems Microbiology, Faculty of Medicine, Chulalongkorn University, Bangkok 10330, Thailand; 3 Department of Biochemistry, Faculty of Medicine, Chulalongkorn University, Bangkok 10330, Thailand; 4 Center of Excellence in Clinical Virology, Faculty of Medicine, Chulalongkorn University, Bangkok 10330, Thailand

**Keywords:** genome characterization, influenza A virus, mutation, next-generation sequencing, Thailand

## Abstract

The influenza A viruses have high mutation rates and cause a serious health problem worldwide. Therefore, this study focused on genome characterization of the viruses isolated from Thai patients based on the next-generation sequencing technology. The nasal swabs were collected from patients with influenza-like illness in Thailand during 2017-2018. Then, the influenza A viruses were detected by reverse transcription-quantitative polymerase chain reaction and isolated by MDCK cells. The viral genomes were amplified and sequenced by Illumina MiSeq platform. Whole genome sequences were used for characterization, phylogenetic construction, mutation analysis and nucleotide diversity of the viruses. The result revealed that 90 samples were positive for the viruses including 44 of A/H1N1 and 46 of A/H3N2. Among these, 43 samples were successfully isolated and then the viral genomes of 25 samples were completely amplified. Finally, 17 whole genomes of the viruses (A/H1N1, n=12 and A/H3N2, n=5) were successfully sequenced with an average of 232,578 mapped reads and 1,720 genome coverage per sample. Phylogenetic analysis demonstrated that the A/H1N1 viruses were distinguishable from the recommended vaccine strains. However, the A/H3N2 viruses from this study were closely related to the recommended vaccine strains. The nonsynonymous mutations were found in all genes of both viruses, especially in HA and NA genes. The nucleotide diversity analysis revealed negative selection in the *PB1*, *PA*, hemagglutinin (*HA*) and neuraminidase (*NA*) genes of the A/H1N1 viruses. High-throughput data in this study allow for genetic characterization of circulating influenza viruses which would be crucial for preparation against pandemic and epidemic outbreaks in the future.

## Introduction

Currently, influenza viruses are still a major cause of respiratory disease and can affect all age groups, resulting in a serious public health problem. The estimated infection rate of influenza viruses is approximately 5% to 15% of the population [1]. Furthermore, there are more than 500,000 deaths reported worldwide [2]. Seasonal influenza is caused by influenza A (A/H1N1 and A/H3N2 subtypes) and influenza B (B/Victoria and B/Yamagata lineages) viruses. However, the influenza A viruses cause more severity, and lead to more epidemics and pandemics due to the high mutation rates which result from antigenic drifts and antigenic shifts [3]. First, antigenic drift is caused by the accumulation of point mutations that change the properties of the viral hemagglutinin (HA) and neuraminidase (NA) surface proteins to avoid the host immune system. On the other hand, an antigenic shift is a genetic reassortment process when at least two strains of influenza A viruses have infected within the same cell [4]. During viral replication, the high rate of mutation is promoted by error-prone polymerase enzyme [5]. The mutation rates of the influenza A virus have been reported within a range of 2.0×10^−6^ to 2.0×10^−4^ mutations per site per round of genome replication [6-8]. Therefore, this evidence suggests that each replicated genome of influenza A carries an average of 2–3 mutations per genome [9]. The virus has gradually adapted to its antigenic sites to avoid the host immune response and vaccination [10]. Due to the high mutation rates, the influenza vaccine was less effective (only 29% to 61%) against seasonal outbreaks during 2019-2020 [11].

In Thailand, influenza transmission occurs year-round with two annual peaks: a major peak in the rainy season and a minor peak in winter [12]. Previous studies have reported that influenza was a major cause of morbidity and mortality in Thailand and resulted in crucial economic costs annually. A study conducted during 2005–2008, estimated an annual average of 36,400 influenza-associated hospitalizations and 300 deaths occurred, with significantly higher mortality rates in children and the elderly [13]. Furthermore, several studies examined the genetic variabilities within HA and NA genes of influenza A viruses based on Sanger sequencing [14-17]. Interestingly, whole genome sequencing (WGS) can be applied to characterize viral strains and provide the comprehensive information of the influenza genome for better understanding of the viral evolution and novel viral strains [18].

Nowadays, next-generation sequencing (NGS) has the advantages of massively parallel sequencing thus making it the ideal tool for characterization of the viral whole genome, viral reassortment and viral mutations [18,19]. Consequently, WGS of influenza viruses based on NGS technology can provide the information necessary to understand the characteristics of influenza viruses. This study aimed to investigate the viral genome and mutations of influenza A viruses circulating in Thailand from 2017 to 2018, and this approach can be further applied for preparation against pandemic and epidemic outbreaks in the future.

## Methods

### Sample collection and influenza diagnosis

The study protocol was approved by the Institutional Review Board (IRB No. 337/57) and Institutional Biosafety Committee (MDCU-IBC No. 001/2018) from the Faculty of Medicine, Chulalongkorn University. Briefly, nasal swab samples from patients with influenza-like illness (ILI) were obtained from Bangpakok 9 International Hospital and Chum Phae Hospital from August 2017 to November 2018. The clinical samples were preserved in viral transport media consisting of Hank’s Balanced Salt Solution supplemented with 1% bovine serum albumin, amphotericin B (15 µg/mL), penicillin G (100 U/mL), and streptomycin (50 µg/mL). The nasal swab samples were screened for influenza virus infection using a one-step multiplex reverse transcription-quantitative polymerase chain reaction (RT-qPCR) as described previously [20,21]. Briefly, the assay was performed in a 10 µL final volume, containing 1 µL of RNA sample, 5 µL of 2× reaction mix, 0.2 µL of SuperScript III RT/Platinum *Taq* Mix (Invitrogen, Carlsbad, CA, USA), an additional 0.1 mM of MgCl_2_, 0.25 µM of each primer, and 0.125 µM of each probe. The one-step multiplex RT-qPCR was performed on the StepOnePlus Real-Time PCR System (Applied Biosystems, Foster City, CA, USA) using the following thermal cycling conditions: at 55°C for 30 min for reverse transcription, followed by 95°C for 10 min, continuing with 40 cycles of 95°C for 15 s and 60°C for 30 s.

### Cell cultures

Madin-Darby canine kidney (MDCK) cells were obtained from the American Type Culture Collection (ATCC, Manassas, VA, USA) and cultured in Dulbecco’s modified Eagle Medium (DMEM) with high glucose (HyClone, Logan, UT, USA) supplemented with 10% fetal bovine serum (Gibco, Grand Island, NY, USA) and 1% (v/v) penicillin/streptomycin (Gibco) maintained under humidified 5% CO_2_ at 37°C [22].

### Influenza virus isolation

MDCK cells were used for influenza virus isolation and propagation as described in the previous study [23]. Briefly, MDCK cells were seeded in 60 mm tissue culture dishes (SPL Life Science, Pocheon, Korea) at 5×10^5^cells per dish in DMEM medium without antibiotics. When the cells reached around 80% confluence, the media were removed and then washed by phosphate buffer saline (PBS) (Amresco, Solon, OH, USA). Positive influenza samples were used for virus isolation. Briefly, 500 µL of a nasal swab from influenza-positive samples was mixed with 500 µL of DMEM with high glucose (HyClone) and filtered through 0.22 µm filter (Millipore, Billerica, MA, USA). The filtrate was immediately processed to influenza viral propagation. Three hundred microliters of each filtered influenza-positive sample were mixed with 200 µL infection medium (DMEM-high glucose supplemented with 2 mM L-glutamine and 0.5 µg/mL TPCK-trypsin). The mixture was added in each dish and then incubated in 5% CO_2_ at 37°C for 1 h. After incubation, the virus suspension was removed and then washed with PBS. Finally, the cells were overlaid with fresh infection medium and incubated under 5% CO_2_ at 37°C for 48 h. After that, the cytopathic effect of infected cells was observed and the viral supernatant was collected. Each sample was isolated in three serial passages (P0‒P2). The viral titers were quantified by RT-qPCR [24] .

### Viral RNA extraction and reverse transcription

One hundred and fifty microliters of the supernatant in each isolation passage were extracted using a GenUp Viral RNA kit (Biotechrabbit, Berlin, Germany) according to the manufacturer’s instructions, and eluted in 60 µL with warm RNase-free water. The concentration of total viral RNA was quantified by using Nanodrop UV spectrophotometer (Implen, Munchen, Germany). Three hundred nanograms per microliter of viral RNA were reverse transcribed into cDNA using the RevertAid First Strand cDNA Synthesis Kit (Thermo Fisher Scientific, Waltham, MA, USA) according to the manufacturer’s instructions with 10 µM reverse transcription primer (5ʹ-ACGCGTGATCAGCAAAAGCAGG-3ʹ) that is conserved and complemented with 12 nucleotides at the 3ʹ ends of each influenza A viral genes [25]. The mixtures were incubated at 42°C for 1.5 h and heat-inactivated at 70°C for 10 min. The cDNAs were stored in ‒20°C for further analysis.

### Quantitative real-time PCR

To determine the amount of influenza virus in each sample passage, the viral matrix (*M*) gene was amplified based on StepOnePlus Real-time PCR Systems (Applied Biosystems) using SYBR Green Luna Universal qPCR Master Mix (New England Biolabs, Ipswhich, MA, USA) as described above. The results were analyzed by StepOnePlus Software v2.3 (Thermo Fisher Scientific). The samples amplified with Ct values lower than 28 were interpreted as positive influenza viral propagation [26].

### Amplifications of influenza genomes

The viral cDNAs from the previous step were used as templates for genome amplifications. The primer sets; forward primer (5ʹ-ACGCGTGATCAGCAAAAGCAGG-3ʹ) and reverse primer (5ʹ-ACGCGTGATCAGTAGAAACAAGG-3ʹ) were used for amplification of influenza A viral genes (8 segments) following the previous study [25]. Briefly, PCR master mix is composed of 1.25 µM of each primer, 0.35 mM of dNTPs, 0.02 U/µL of Phusion High-Fidelity DNA polymerase (Thermo Fisher Scientific), 7.5 µL of cDNA, and nuclease-free water to a final volume of 50 µL. Subsequently, 15 µL of the amplicons were analyzed by 1% agarose gel electrophoresis. The amplicons were purified by the QIAquick PCR Purification kit (Qiagen, Hilden, Germany) following the manufacturer’s protocol. The concentrations of purified PCR products were measured by the Qubit dsDNA High-Sensitivity assay kit (Invitrogen).

### DNA library preparation and next-generation sequencing

The purified amplicons (1 µg in 130 µL) from the genome amplification step were sheared to approximately 200 bp fragments by the Covaris M220 Focused-ultrasonicator (Covaris, UK) with 20% duty factor, 50 unit of peak incident power (W), 200 cycles per burst for 150 s. The fragmented DNAs were used for DNA library preparation by using NEBNext Ultra II DNA Library Prep Kit for Illumina (New England Biolabs) following the manufacturer’s instructions. Briefly, 50 µL of DNA fragments were ends repaired and subsequently adapters ligated by using the NEB ligase master mix. Then, DNA libraries (approximately 320 bp) were cleaned up and size selected by AMPure XP beads (Beckman Coulter, Brea, CA, USA). For library enrichment, PCR amplification was carried out by adding the Illumina MiSeq-compatible indexes to the DNA libraries. Afterwards, the enriched DNA libraries were purified by 2% agarose gel electrophoresis with 100 V for 20 min and size selected (approximately 320 bp). Finally, the total DNA libraries were quantified by real-time PCR using the KAPA Library Quantification Kits (Kapa Biosystems, Wilmington, MA, USA). After that, the concentration of each sample was determined and pooled equally at 2 nM of each library. Subsequently, the pooled library was then diluted to 6 pM and paired-end sequenced (2×150 bp) on an Illumina MiSeq instrument using MiSeq Reagent Kits v2 (300 cycles) according to the manufacturer protocol (Illumina).

### Influenza genome analysis

The MiSeq Reporter Software version 2.4 was used for the primary analysis of FASTQ sequencing data. Low-quality reads (Q-score <30) and adaptors were trimmed. The passing filtered reads (Q-score ≥30) were aligned with the vaccine strains of influenza A reference genomes (A/California/07/2009 (H1N1) or A/South Australia/55/2014 (H3N2)) for genome characterization and mutation analysis by using CLC Genomics Workbench software (Qiagen). Mutation patterns and frequencies were generated by using GraphPad Prism version 6.01 software (GraphPad Software Inc., San Diego, CA, USA). The FASTQ files and FASTA files of influenza genome sequences were submitted to the Sequence Read Archive (BioProject ID: PRJNA576776) and GenBank as shown in Supplementary Table 1.

### Phylogenetic analysis

In this study, the HA and NA deduced amino acid sequences of A/H1N1 and A/H3N2 were aligned with reference strains retrieved from the Global Initiative on Sharing All Influenza Data (GISAID) EpiFlu database by using the Clustal W program, implemented in the BioEdit sequence alignment editor software v.7.2.5 [27]. Phylogenetic analysis was performed by mean of the maximum likelihood method (1,000 bootstrapping replicates) and LG with Freqs.(+F) model (discreate gamma distribution with 5-rate categories and complete deletion data subset) using the MEGA X software [28].

### Sliding windows analysis of nonsynonymous nucleotide variation

The nucleotide diversity (π) within each gene of influenza A/H1N1 and A/H3N2 viruses was evaluated by PoPoolation v.1.2.2 to investigate the genetic variations of viruses within the sample [29]. The sliding window analysis of nonsynonymous nucleotide variation (πN) was performed based on Syn-nonsyn-sliding.pl script with the window size of nine codons and a step size of one codon. The average corresponding πN values were calculated and plotted to a middle position of the windows to demonstrate the degree of nonsynonymous substitutions within eight viral gene segments. In addition, the nonsynonymous nucleotide variations (πN) per synonymous nucleotide variation (πS) were analyzed by the Syn-nonsyn-at-position.pl script to investigate the neutrality of selection in each segment. The πN/πS ratios per gene in each influenza subtype were calculated as the average value from individual samples. Lastly, a paired Wilcoxon signed-rank test (p < 0.05) was used to compare pooled average πN and πS values within each subtype of influenza viruses.

## Results

### Detection and isolation of influenza A viruses

In this study, 500 nasal swab samples were collected from patients with ILI and detected for influenza A virus by RT-qPCR. Ninety samples were influenza A virus-positive samples including 48.9% (44 samples) of A/H1N1 and 51.1% (46 samples) of A/H3N2 as shown in Table 1. Among these 90 samples, 43 samples (29 of A/H1N1 and 14 of A/H3N2) were successfully isolated in the second passage (P2) of MDCK cells with Ct value ranging from 13 to 28 (Table 2).

### WGS and characterization of influenza A viruses

From 43 isolated samples, 25 samples were completely amplified as a full genome including 17 samples of A/H1N1 and eight samples of A/H3N2. Finally, 17 samples (12 samples of A/H1N1 and five samples of A/H3N2) passed the quality control of libraries preparation for NGS as shown in Table 1. The result revealed that 17 whole genomes of influenza A viruses were successfully sequenced with an average of 424,151 total reads per sample, 232,578 mapped reads per sample, and 1,720 genome coverage per sample (Table 2). Therefore, these results were highly confident for genome annotation and mutation analysis. The FASTQ data were deposited as BioProject accession no. PRJNA576776 and influenza genome sequences were submitted into GenBank database as summarized in Supplementary Table 1.

### Phylogenetic analysis of influenza A viruses in Thailand

**Fig. 1.**
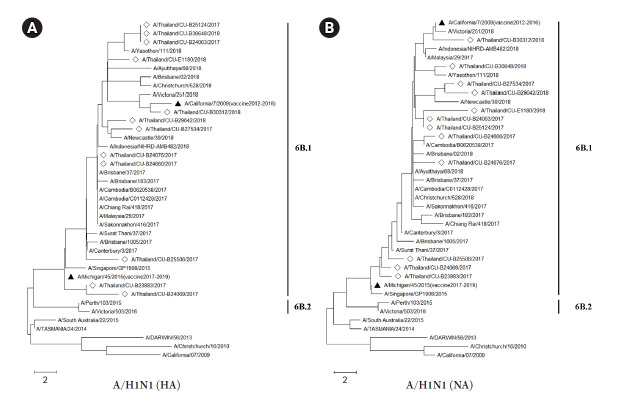
The phylogenetic analysis of influenza A viruses (H1N1) circulating in Thailand during 2017‒2018 (diamond) compared with several World Health Organization recommended influenza vaccine strains (black triangle). The hemagglutinin (HA) (A) and neuraminidase (NA) (B) deduced amino acid sequences were analyzed based on mean of maximum likelihood with 1,000 bootstrapping and LG with Freqs. (+F) model (discreate gamma distribution with 5-rate categories and complete deletion data subset).

**Fig. 2.**
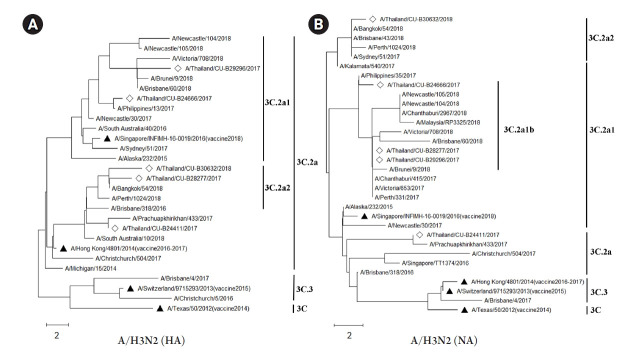
The phylogenetic analysis of influenza A viruses (H3N2) circulating in Thailand during 2017‒2018 (diamond) compared with several WHO recommended influenza vaccine strains (black triangle). The hemagglutinin (HA) (A) and neuraminidase (NA) (B) deduced amino acid sequences were analyzed based on maximum likelihood with 1,000 bootstrapping and LG with Freqs. (+F) model (discreate gamma distribution with 5-rate categories and complete deletion data subset).

The HA and NA deduced amino acid sequences were used for phylogenetic analysis. The sequences were aligned with the HA and NA deduced amino acid sequences of the influenza vaccine strains (southern hemisphere influenza seasons during 2012‒2019) recommended by the World Health Organization (WHO). The influenza A/H1N1 viruses isolated from this study during 2017-2018 belonged to genetic subclade 6B.1. Interestingly, the results demonstrated that A/H1N1 viruses were closely related to influenza (A/California/7/2009) strain and distinguishable from the recommended influenza vaccine strains for use in 2017-2019 (A/Michigan/45/2015 (H1N1)) as shown in Fig. 1A and 1B. On the other hand, the circulating A/H3N2 strains, classified into subclade 3C.2a1 and 3C.2a2, were comparatively more closely related to the 2017‒2018 WHO influenza vaccine strains (A/Hong Kong/4801/2014 (H3N2) and A/Singapore/INFIMH-16-0019/2016 (H3N2)) as shown in Fig. 2A and 2B.

### Nucleotide diversity of influenza A viruses

**Fig. 3.**
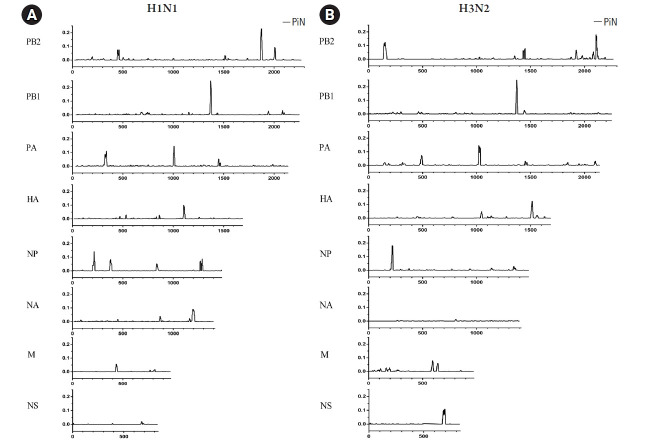
Sliding windows analysis of nonsynonymous nucleotide variation (πN) in eight genes of influenza A virus subtypes H1N1 (A) and H3N2 (B). The πN values were determined by sliding windows with the window size of nine codons and a step size of one codon. The mean corresponding πN values were calculated and plotted to a middle site of the windows.

The variations of nonsynonymous within influenza A viruses among the samples in this study are summarized in Fig. 3. As shown in Fig. 3A, strong signals appeared in the polymerase (*PB2*, *PB1*, and *PA*) genes as well as in the *NP* gene in A/H1N1 virus. However, the NA, *M*, and *NS* genes showed the low nonsynonymous variations. Interestingly, the HA gene contained the pattern of the variation signals around the middle position of this A/H1N1 gene. As for the results of A/H3N2 (Fig. 3B), the polymerase genes were presented as sharp and multiple peaks of the nonsynonymous nucleotide diversity. In addition, the HA, *NP*, and NA genes of A/H3N2 displayed sharp signals at the beginning and the end of these genes. Furthermore, the *M* and *NS* genes of the A/H3N2 only had peaks around the middle of the genes.

**Fig. 4.**
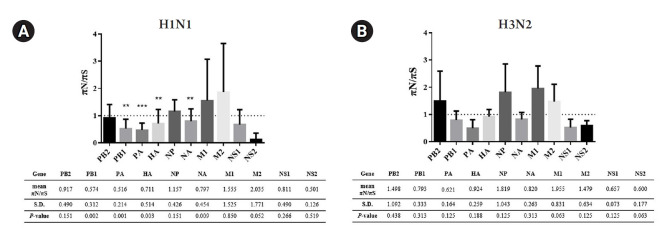
The ratio of nonsynonymous nucleotide variation (πN) to synonymous nucleotide variation (πS) analysis in eight genes of influenza A virus subtypes H1N1 (A) and H3N2 (B). Significant at ^*^p < 0.05, ^**^p < 0.01 and ^***^p < 0.001. The ratio πN/πS > 1: positive selection; πN/πS < 1: negative selection; πN/πS ≈ 1: neutral evolution. The mean πN/πS, standard deviation (S.D.), and p-value (Student’s t-test) in each segment were summarized at the bottom of the figure.

Exploring deeper detail about the direction of diversity, the ratios of nonsynonymous to synonymous nucleotide diversity (πN/πS) were introduced to examine the changes in nucleotide variations. In brief, the πN/πS ratios > 1 indicate that selective pressure promotes the new variations (positive selection). In contrast, the πN/πS ratios < 1 refer to the new variation being unfavored (negative selection). In addition, the πN/πS ratios ≈ 1 suggest that neutral evolution occurs in these new variations. According to the results shown in Fig. 4, there was no significant positive selection occurring in this study. However, the statistically significant negative selections (p < 0.05) were found in *PB1*, *PA*, HA, and NA genes of A/H1N1. Meanwhile, the A/H3N2 exhibited random selections within these 10 genes due to there being no significant difference observed in the πN/πS ratios.

## Discussion

In this study, 90 out of 500 (18%) nasal swabs obtained from Thai patients with influenza-like-illness during 2017‒2018 were positive for influenza A virus detection based on RT-qPCR detection. The percentage of influenza A virus positive in this research was slightly higher than those reported in the previous study (13.2%) during 2016‒2017 in Thailand [30]. Previously, several studies have demonstrated that the appropriate quality and quantity of DNA are important for the successful NGS platform sequencing [31-33]. In particular, this study has successfully isolated 47.78% (43 of 90 samples) which are positively identified as the influenza A virus, which is higher than the previous study (3.04% of isolation rate) [34]. Also, the positive virus isolations (58.14%, 25 of 43 isolates) can be amplified with universal primers, following the study of Meinel et al. [35], which is appropriate for whole genome characterization and mutation analysis of influenza A virus. For NGS analysis, the result revealed that 17 whole genomes of influenza viruses were successfully sequenced with 232,578 mapped reads (424,151 total reads), average read length of 96.3 bp and average 1,720.4 genome coverage. Furthermore, the complete sequences of the viral genomes provide reliable and highly informative data despite the average genome coverages, depth coverage, which ranged from 237.7 to 4,229.7 (Table 2). The advantages of the NGS-based technique are that it provides the full genome segment and whole genome of influenza virus, as well as effectively reducing both the turnaround time and cost per nucleotide sequence for the whole genome when compared to the Sanger sequencing method [36-38]. However, the sequencing with the Sanger method does not provide the data for quasispecies and nucleotide diversity analysis. Interestingly, the NGS provides more information for minor mutations and selection pressures within the viral genome. Indeed, the nucleotide variations obtained from NGS can be applied for calculation of viral nucleotide diversity within each sample [39,40].

The number of mutations in the HA and NA genes of A/H1N1 might affect the efficiency of a vaccine, and related to deduced amino acid sequences of phylogenetic tree (Fig. 1A and 1B). The vaccine effectiveness of the 2017‒2018 flu vaccine against both influenza A viruses is approximately 25% to 52% in Europe and 27% to 44% in the United States [41,42]. Interestingly, the result of the influenza A/H1N1 phylogenetic tree with deduced amino acid sequences, which belongs to clade 6B.1, showed a long distance between vaccine strains for 2017‒2018 (A/Michigan/42/2015) and our A/H1N1 samples. This result implied that the vaccine might be less effective against A/H1N1 in Thailand. Moreover, the report from the US Centers for Disease Control and Prevention (CDC) also showed the vaccine effectiveness against A/H1N1 was 65% [43]. However, the phylogenetic analysis of both HA and NA deduced amino acid sequences revealed the closer relationship between A/H3N2 isolates (clade 3C.2a1 and 3C.2a2) and A/Hong Kong/4801/2014 strain which was the recommended vaccine virus for A/H3N2 [44]. Therefore, these results implied that the recommended vaccine was more effective against the influenza A/H3N2 in Thailand during 2017‒2018. Indeed, the phylogenetic trees of influenza A/H1N1 and A/H3N2 obtained in this study were correlated with recent genetic and antigenic characterizations of influenza viruses in Thailand [45].

**Fig. 5.**
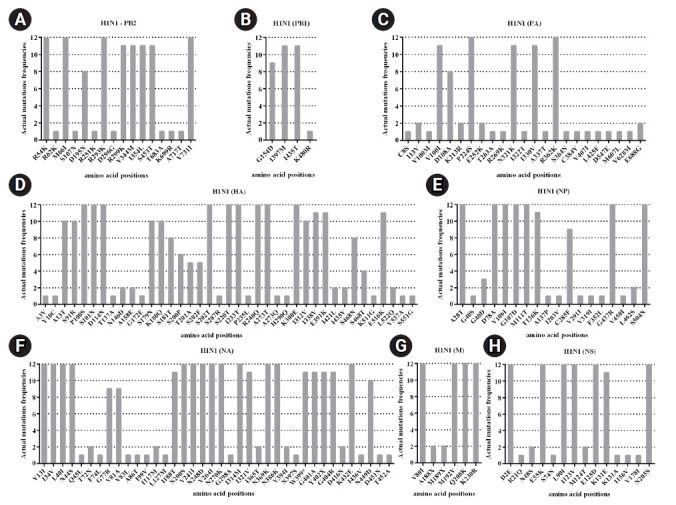
Mutation patterns with actual mutations frequencies observed in each viral gene segments of influenza A virus (H1N1): (A) PB2, (B) PB1, (C) PA, (D) HA, (E) NP, (F) NA, (G) M, and (H) NS. Amino acid changes were compared to the reference sequence (A/California/07/2009 (H1N1)).

In this study, the genome of circulating influenza A viruses in Thailand during 2017‒2018 was characterized. The result from NGS analysis not only provided the full genome of the virus but also acquired the amino acid substitutions across eight segmented genes. Moreover, there were several known functional mutations of influenza A/H1N1 that had been already characterized. Firstly, the mutations at I354, V344M, and S453T in the PB2 (Fig. 5A) could regulate in the cap-snatching from host RNAs during the viral RNA transcription process [46]. Furthermore, N321K in the PA was reported to increase the polymerase complex activity and the viral replication in the cell culture [46,47]. The amino acid substitution at V100I in the PA-X could trigger down-regulated innate immune response genes (Fig. 5C). Indeed, the amino substitutions at S91R, S181T, I312V, and E391K in the HA might be related to adaptive genetic variations that alter the salt bridge pattern and the membrane fusion stability for major antigenic sites and glycan specificity [46,47]. Three mutations (K180Q, S202T, and S220T) were located in the HA antigenic sites, which might be involved in the pathogenicity and contributed to the epidemic [48]. Moreover, the mutation (S220T) was observed to affect the infectivity and transmissibility of the virus in humans [49]. The mutation (R240Q) was found in the receptor-binding domain of the HA, which has been reported to increase virus growth [50]. The amino acid variations (D114N, K180Q, S202T, S220T, and K300E) were responsible for loss of antibody neutralization and decreased overall vaccine effectiveness (Fig. 5D) [51-53]. The amino acid substitutions (N44S, V241I, and N369K) in the NA have been reported to facilitate the stability of the virus [54]. The I188T and N449D mutations in the NA found in this study are similar to those reported in the previous study [55]; however, the function of the mutations has not been well characterized (Fig. 5F). Additionally, the nonsynonymous mutation at E55K, L90I, I123V, E125D, K131E, and N205S in the NS1 involves the inhibition of host gene expressions related to the interferon response [56,57]. Indeed, the E125D mutation in NS1 (Fig. 5H) interacts with cellular cleavage and polyadenylation specificity factor 30 (CPSF30), which is considered potential in host adaptation to influenza A/H1N1 virus [58,59].

**Fig. 6.**
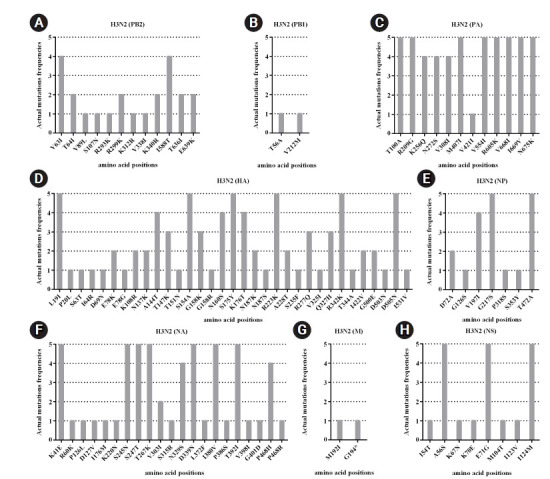
Mutation patterns with actual mutations frequencies observed in each viral gene segments of influenza A virus (H3N2). Amino acid changes were compared to the reference sequence (A/South Australia/55/2014 (H3N2)).

In the influenza A/H3N2, the previous reports found R277Q and D69N at the antigenic epitope C, N137K, and N187K at the antigenic epitope D and E78K/G at the antigenic epitope E of the HA [60,61]. Among these, four amino acid substitutions (N137K, N187K, I422V, and G500E) belong to clade 3C.2a.1 and are represented by A/Singapore/INFIMH-16-0019/2016(H3N2) virus [62]. The T151N substitution in HA protein was related to the potential N-glycosylation site, affecting antigenic and other viral properties. Moreover, the Q327H substitution in the HA was suggested to bind host proteins (Fig. 6D) [62]. Since 2016, the accumulation of mutation at S245N of the NA has contributed to an N-glycosylation site. These mutations (S245N, S247T, and P468H) were introduced to the NA antigenic drift of the circulating A/H3N2 virus [63]. However, N329S mutation could result in a loss of N-glycosylation in the NA [64]. The V303I substitution has been observed in the NA protein (Fig. 6F) with a low resistance to NA inhibitors [65]. Indeed, most mutations of influenza A viruses observed in this study were identified as novel mutations which have not been reported yet (Figs. 5 and 6). However, the function of the novel mutations needs to be further investigated.

Nonsynonymous (πN) and synonymous (πS) mutations of the viruses can be accessed by NGS leading to nucleotide diversity (πN/πS) analysis. According to the previous study, the deep sequencing of A/Wisconsin/67/2005 (H3N2) revealed that the positive selection was observed in the viruses isolated from the chicken kidney, Vero cell culture, and embryonated chicken eggs, whereas the negative selection was found in virus from direct intranasal inoculation in the human challenge [40]. There was no significant nucleotide diversity observed in A/H3N2 viruses in our study, and this might be due to the strain of the virus, host cell, or limited numbers of the sample. For πN/πS analysis of influenza A/H1N1 viruses, the mutations existing in the viral genes with statistical significance were *PB1*, *PA*, HA, and NA genes in which these mutations were suggested as negative selection. Therefore, to further investigate the πN and πS variations, the sliding window analysis of those genes was performed to ensure that the negative selections were not the outcome of the averaging value across the entire gene. The results of sliding window analysis were consistent with the negative selections from the πN/πS analysis in those genes at which the πN signals were high and sharp at some regions of the genes, while the rest of the genes were relatively low in the πN signals.

In summary, the NGS was successfully applied for whole genome characterizations of influenza A/H1N1 and A/H3N2 viruses that provide the high-throughput data for phylogenetic construction, mutation analysis, and nucleotide diversity. The results revealed that the recommended vaccine A/H1N1 strain might be less effective against the A/H1N1 virus. Moreover, several mutations were demonstrated in both A/H1N1 and A/H3N2, especially in HA and NA genes. Finally, the negative selections were found in the *PB1*, *PA*, HA, and NA genes of the A/H1N1. Unfortunately, limited number of samples were successfully propagated, amplified, and sequenced in this study. Nevertheless, the whole genome data obtained from this study might be useful for mutation analysis and can be compared with data obtained from other studies in the future.

## Figures and Tables

**Table 1.** The amount of positive influenza A samples obtained from RT-qPCR, virus isolation, genome amplification, and NGS

	Positive samples	Positive virus isolation	Genome amplification	NGS
Influenza A/H1N1	44	29	17	12
Influenza A/H3N2	46	14	8	5
Total	90	43	25	17

RT-qPCR, reverse transcription-quantitative polymerase chain reaction; NGS, next-generation sequencing.

**Table 2.** The sample characteristic, virus isolation and NGS data of influenza A virus in this study

No.	Sample name	Age (yr)	Sex	Ct from each passage			Total reads	Mapped reads	Average length of mapped read (bp)	Average genome coverage	
				P0	P1	P2				
1	A/Thailand/CU-B23883/2017 (H1N1)	2	M	38	30	27	318,034	54,644	67.2	237.7	
2	A/Thailand/CU-B24063/2017 (H1N1)	19	F	46	34	17	171,064	163,257	82.4	905.6	
3	A/Thailand/CU-B24069/2017 (H1N1)	39	M	36	23	18	325,424	309,926	84.4	1,784.8	
4	A/Thailand/CU-B24076/2017 (H1N1)	12	M	36	33	28	143,698	134,494	79.6	726.3	
5	A/Thailand/CU-B24660/2017 (H1N1)	51	F	33	18	16	518,852	442,915	96.0	2,907.9	
6	A/Thailand/CU-B25124/2017 (H1N1)	3	M	31	32	20	134,102	129,280	87.8	771.9	
7	A/Thailand/CU-B25506/2017 (H1N1)	38	F	32	27	18	165,684	159,654	88.7	952.3	
8	A/Thailand/CU-B27534/2017 (H1N1)	31	F	28	13	13	124,720	112,724	61.6	477.5	
9	A/Thailand/CU-B29642/2018 (H1N1)	30	F	32	29	16	1,355,512	520,427	113.6	3,927.2	
10	A/Thailand/CU-B30312/2018 (H1N1)	59	F	31	27	27	199,458	180,341	120.6	1,460.6	
11	A/Thailand/CU-B30648/2018 (H1N1)	29	F	33	22	15	702,634	367,078	113.6	2,771.4	
12	A/Thailand/CU-E1180/2018 (H1N1)	2	M	20	30	13	555,488	357,666	100.3	2,351.7	
13	A/Thailand/CU-B24411/2017 (H3N2)	61	F	34	26	23	217,002	68,933	60.0	284.1	
14	A/Thailand/CU-B24666/2017 (H3N2)	2	F	27	31	15	139,802	90,676	112.1	696.8	
15	A/Thailand/CU-B28277/2017 (H3N2)	24	M	23	19	24	583,222	467,163	129.5	4,229.7	
16	A/Thailand/CU-B29296/2017 (H3N2)	52	F	30	23	22	557,710	22,569	132.3	2,013.4	
17	A/Thailand/CU-B30632/2018 (H3N2)	53	M	31	24	24	998,160	372,082	107.3	2,747.7	
	Average						424,151	232,578	96.3	1,720.4	

NGS, next-generation sequencing.

## Supplementary Materials

Supplementary Table 1. The summary of accession numbers of influenza A virus in this study
