# Supplementary material for: Genome characterization and mutation analysis of human influenza A virus in Thailand
Source: Genomics Inform. 2022 Jun 30;20(2):e21. doi: 10.5808/gi.21077 (PMC9299564; doi:10.5808/gi.21077)
Supplement: Supplementary Table 1. — The summary of accession numbers of influenza A virus in this study [file gi-21077suppl.pdf]

Supplementary Table 1. The summary of accession numbers of influenza A virus in this study

Strain name	SRA accession No. FASTQ data	FASTA accession No.							
		PB2	PB1	PA	HA	NP	NA	M	NS
A/Thailand/CU-B23883/2017(H1N1)	SRR10256725	MN560613	MN560614	MN560615	MN560616	MN560617	MN560618	MN560619	MN560620
A/Thailand/CU-B24063/2017(H1N1)	SRR10256724	MN560778	MN560779	MN560780	MN560781	MN560782	MN560783	MN560784	MN560785
A/Thailand/CU-B24069/2017(H1N1)	SRR10256713	MN560975	MN560976	MN560977	MN560978	MN560979	MN560980	MN560981	MN560982
A/Thailand/CU-B24076/2017(H1N1)	SRR10256710	MN561022	MN561023	MN561024	MN561025	MN561026	MN561027	MN561028	MN561029
A/Thailand/CU-B24660/2017(H1N1)	SRR10256709	MN561043	MN561044	MN561045	MN561046	MN561047	MN561048	MN561049	MN561050
A/Thailand/CU-B25124/2017(H1N1)	SRR10256708	MN561055	MN561056	MN561057	MN561058	MN561059	MN561060	MN561061	MN561062
A/Thailand/CU-B25506/2017(H1N1)	SRR10256707	MN561178	MN561179	MN561180	MN561181	MN561182	MN561183	MN561184	MN561185
A/Thailand/CU-B27534/2017(H1N1)	SRR10256706	MN561198	MN561199	MN561200	MN561201	MN561202	MN561203	MN561204	MN561205
A/Thailand/CU-B29642/2018(H1N1)	SRR10256705	MN561252	MN561253	MN561254	MN561255	MN561256	MN561257	MN561258	MN561259
A/Thailand/CU-B30312/2018(H1N1)	SRR10256704	MN561279	MN561280	MN561281	MN561282	MN561283	MN561284	MN561285	MN561286
A/Thailand/CU-B30648/2018(H1N1)	SRR10256723	MN561288	MN561289	MN561290	MN561291	MN561292	MN561293	MN561294	MN561295
A/Thailand/CU-E1180/2018(H1N1)	SRR10256722	MN561304	MN561305	MN561306	MN561307	MN561308	MN561309	MN561310	MN561311
A/Thailand/CU-B24411/2017(H3N2)	SRR10256721	MN561330	MN561331	MN561332	MN561333	MN561334	MN561335	MN561336	MN561337
A/Thailand/CU-B24666/2017(H3N2)	SRR10256720	MN561322	MN561323	MN561324	MN561325	MN561326	MN561327	MN561328	MN561329
A/Thailand/CU-B28277/2017(H3N2)	SRR10256719	MN561350	MN561351	MN561352	MN561353	MN561354	MN561355	MN561356	MN561357
A/Thailand/CU-B29296/2017(H3N2)	SRR10256718	MN561279	MN561280	MN561281	MN561282	MN561283	MN561284	MN561285	MN561286
A/Thailand/CU-B30632/2018(H3N2)	SRR10256717	MN560639	MN560640	MN560641	MN560642	MN560643	MN560644	MN560645	MN560646
